# Supplementary material for: Impact of in-hospital discontinuation with angiotensin receptor blockers or converting enzyme inhibitors on mortality of COVID-19 patients: a retrospective cohort study
Source: BMC Med. 2021 May 12;19:118. doi: 10.1186/s12916-021-01992-9 (PMC8114973; doi:10.1186/s12916-021-01992-9)
Supplement: Supplementary file 1 — Additional file 1. Figures S1-S8 and Tables S1-S5. [file 12916_2021_1992_MOESM1_ESM.pdf]

ADDITIONAL FILE 1

IMPACT OF IN-HOSPITAL DISCONTINUATION WITH ANGIOTENSIN RECEPTOR BLOCKERS OR ANGIOTENSIN CONVERTING ENZYME INHIBITORS ON MORTALITY OF COVID-19 PATIENTS

A RETROSPECTIVE COHORT STUDY

Prof Francisco J. de Abajo, MD, PhD, MPH[1,2], Antonio Rodríguez-Miguel, PhD[1,2], Sara Rodríguez-Martín, PhD[1,2], Victoria Lerma, RN[1], Alberto García-Lledó, MD, PhD[3,4], on behalf of MED-ACE2-COVID19 Study Group*

1. Clinical Pharmacology Unit. University Hospital Príncipe de Asturias. Alcalá de Henares. Madrid. Spain
2. Department of Biomedical Sciences (Pharmacology Section). University of Alcalá (IRYCIS). Alcalá de Henares. Madrid. Spain
3. Department of Cardiology, University Hospital Príncipe de Asturias. Alcalá de Henares. Madrid. Spain
4. Department of Medicine, University of Alcalá (IRYCIS). Alcalá de Henares. Madrid. Spain.

Short title: In-hospital use of RAS inhibitors and mortality in COVID-19 patients

Key words: Renin-angiotensin system inhibitors, angiotensin-converting enzyme inhibitors, angiotensin receptor blockers, COVID-19, mortality, in-hospital treatment

Author for correspondence:
Prof. Francisco J. de Abajo
Clinical Pharmacology Unit
University Hospital Principe de Asturias
Department of Biomedical Sciences, University of Alcalá (IRYCIS)
Tel. 91887 81 00 (ext 2607) - 91 885 25 93
e-mail: francisco.abajo@uah.es

***MED-ACE2-COVID19 Study Group:**
Hospital Universitario Príncipe de Asturias: F J de Abajo, A Rodríguez-Miguel, S Rodríguez-Martín, V Lerma, A García-Lledó, D Barreira-Hernández; D Rodríguez-Puyol; *Hospital Universitario de Getafe*: O Laosa, L Pedraza, L Rodríguez-Mañas; *Hospital Universitario Ramón y Cajal:* M Aguilar, I de Pablo, MA Gálvez; *Hospital Central de la Defensa Gómez Ulla*: A García-Luque, M Puerro; RM Aparicio, V García-Rosado, C Gutiérrez-Ortega; *Hospital Clínico San Carlos*: L Laredo, E González-Rojano, C Pérez, A Ascaso, C Elvira; *Hospital Universitario de La Princesa*: G Mejía-Abril, P Zubiaur, E Santos-Molina, E Pintos-Sánchez, M Navares-Gómez; F Abad-Santos; *Hospital Universitario Puerta de Hierro-Majadahonda*: G A Centeno, A Sancho-Lopez, C Payares-Herrera, E Diago-Sempere.

Figure S1. Relationship between the severity score and in-hospital mortality. Adjusted-hazard ratios of severity score and in-hospital death were obtained trough a regression Cox model after adjusting for age, sex, baseline characteristics, outpatient treatments, hospital and date of admission. Scores 0 and 1, as well as 6 and 7 were collapsed to assure enough number of patients. Test for trend, p=0.01

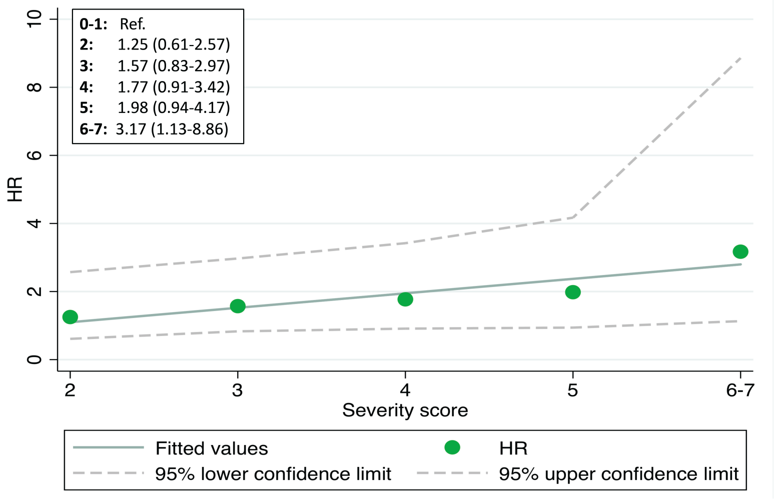

Figure S2: **Hypothesized causal graphs.** X: Discontinuation of RASIs; P: Propensity scores; Z: mediators; Y: outcome; and U: mediator-outcome confounder. The arrow connecting X with Y represents the direct effect of X on Y. The pathway through Z is the indirect effect of X on Y (dashed line). In **a)** it is shown the causal diagram when the estimated effect of X on Y is conditioned (indicated by the square) on the propensity scores (adjustment at once for the set of covariates included in the PS model), but no control for Z or adjustment for U is made; so, we would be estimating the total effect of X on Y, including the effect mediated by Z (additionally confounded by U); in **b)** the estimated effect of X on Y is conditioned on P and Z (controlled by the mediator), but the collider on Z opens a path through U and introduces confounding; in **c)** the effect of X on Y is conditioned on P, Z and U, and the direct effect is estimated by controlling for the mediator and adjusting for the mediator-outcome confounder.

a)

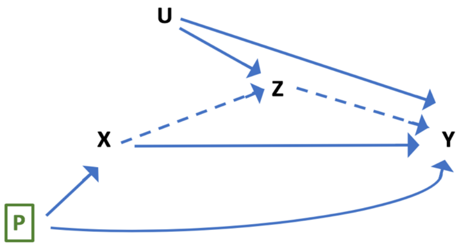

b)

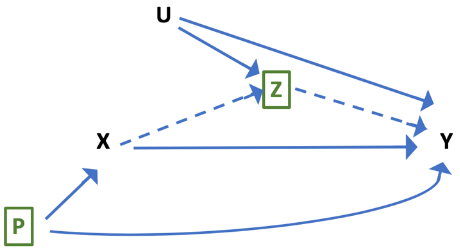

c)

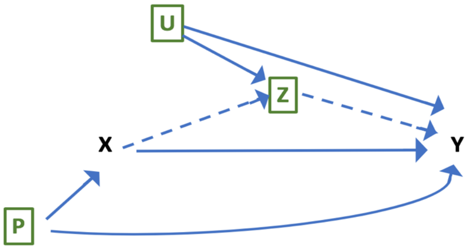

Figure S3. Definition of discontinuation/continuation of RASIs using: a) using a 3-days window (main analysis); **b)** using a 2-days window (sensitivity analysis)

a)

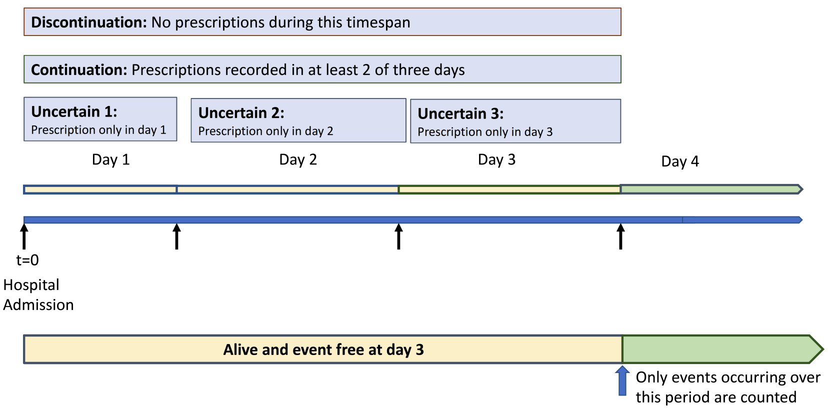

b)

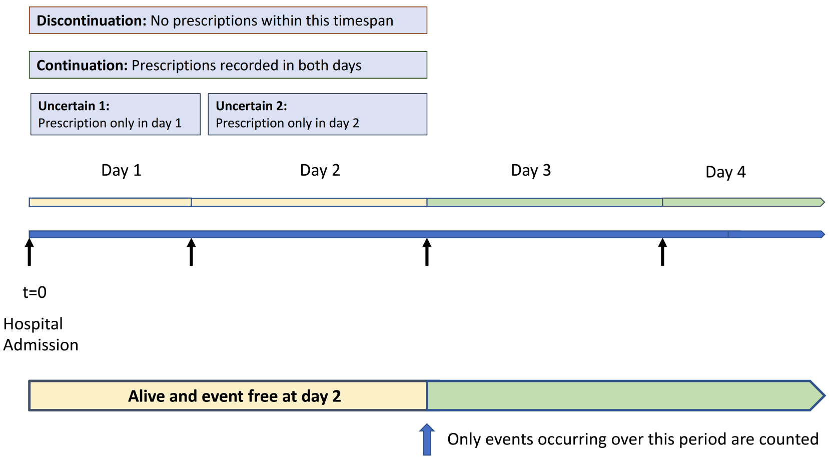

Figure S4: In-hospital use of RASIs by date-of-admission cohort. Patients were grouped in three cohorts according to the date of admission: P1) from 1st to 10th of March (n=81); P2) from 11th to 20th of March (N=416); and P3) from 21st to 31st of March (N=128). The discontinuation rate of RASIs was highly influenced by date of admission. The inpatient use of RASIs remained low during hospital stay in all three cohorts.

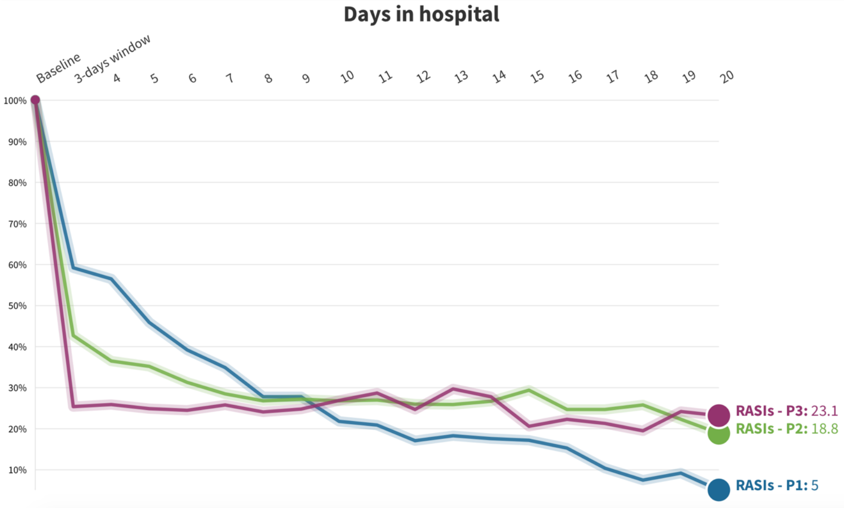

Abbreviations: RASIs: renin-angiotensin system inhibitors
Dynamic visualization available in: https://public.flourish.studio/visualisation/4863807/

Código de campo cambiado

Figure S5: Switching from ACEIs or ARBs to CCBs and other antihypertensive drugs during the first 3 days since hospital admission (patients with uncertain (dis)continuation are excluded). Of all outpatient ARB users, 42.7% continued with ARBs (alone or combined with CCBs or OADs), 31.7% were switched to CCBs or OADs, and 25.6% were left without any antihypertensive treatment. Of all outpatient ACEI users, 44.5% continued with ACEIs (alone or combined with CCBs or OADs), 28.8% were switched to CCBs or OADs, and 26.8% were left without any antihypertensive treatment.

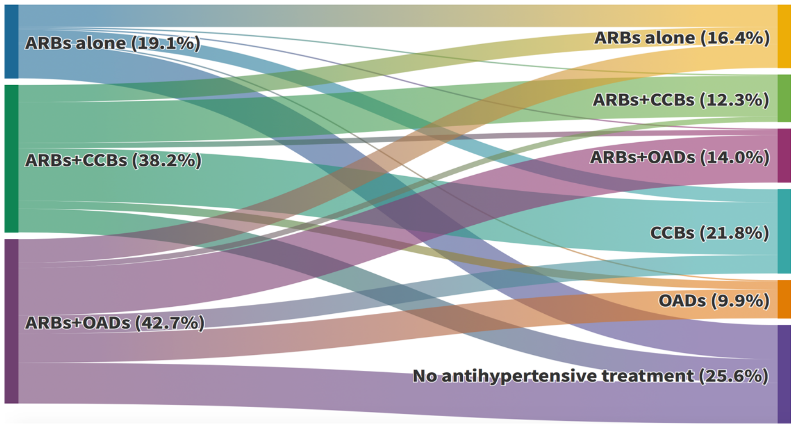

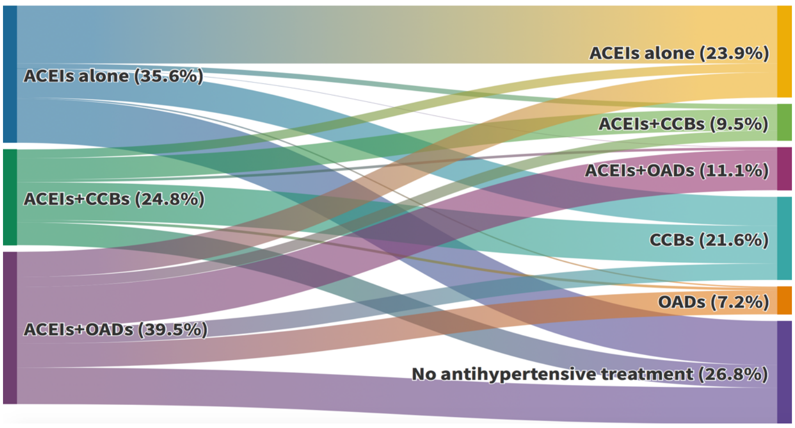

Abbreviations: CCBs: calcium-channel blockers; OADs: other antihypertensive drugs (different from ARBs/ACEIs or CCBs); ARBs: angiotensin receptor blockers; ACEIs: angiotensin-converting enzyme inhibitors

ARBs/ACEIs+CCB: combined use with OADs allowed; **ARBs/ACEIs+OADs:** use of CCBs excluded; **CCBs:** alone or combined with OADs and ARBs/ACEIs excluded; **OADs:** use of ARBs/ACEIs and CCBs excluded.

Dynamic visualizations available in: ARBs: https://public.flourish.studio/visualisation/4886927/
ACEIs: https://public.flourish.studio/visualisation/4887174/

Código de campo cambiado

Figure S6. Forest plots showing mortality rates and adjusted hazard ratios of in-hospital death associated with renin-angiotensin system inhibitors discontinuation vs continuation, by different subgroups (the category of reference is RASIs continued).

	RASIs discontinued Deaths (%)	RASIs continued Deaths (%)	MC–HR (95%CI)*	Test of interaction p–value
**RISK FACTORS:**				
**GENDER**				
Females	36 (28.4)	28 (23.5)	0.96 (0.53–1.76)	0.73
Males	58 (27.2)	51 (30.7)	1.10 (0.69–1.76)	
**AGE (years)**				
< 75	21 (12.3)	19 (13.8)	1.25 (0.59–2.66)	0.50
75+	73 (43.2)	60 (40.8)	0.93 (0.61–1.44)	
**OBESITY**				
No	74 (26.2)	57 (28.6)	0.91 (0.60–1.38)	0.24
Yes	20 (35.1)	22 (25.6)	1.60 (0.74–3.44)	
**DIABETES**				
No	55 (25.4)	46 (26.4)	0.92 (0.58–1.47)	0.69
Yes	39 (31.7)	33 (29.7)	1.07 (0.60–1.92)	
**HEART FAILURE**				
No	80 (25.7)	58 (23.7)	1.07 (0.70–1.61)	0.38
Yes	14 (48.3)	21 (52.5)	0.71 (0.31–1.59)	
**BACKGROUND CV RISK**				
CV risks factors	49 (21.7)	40 (23.8)	0.85 (0.51–1.41)	0.33
CV diseases	45 (39.5)	39 (33.3)	1.22 (0.72–2.07)	
**SEVERITY SCORE**				
0 – 3	50 (23.4)	43 (22.2)	1.06 (0.64–1.77)	0.88
4 – 7	44 (34.9)	36 (39.6)	1.00 (0.58–1.73)	
**IN–HOSPITAL USE OF:**				
**CORTICOSTEROIDS**				
No	43 (24.9)	36 (20.8)	1.19 (0.69–2.06)	0.32
Yes	51 (30.5)	43 (38.4)	0.82 (0.50–1.35)	
**BETA BLOCKERS**				
No	72 (25.7)	56 (25.9)	0.87 (0.57–1.33)	0.39
Yes	22 (36.7)	23 (33.3)	1.26 (0.61–2.59)	

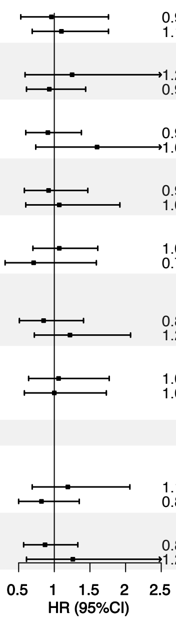

Abbreviations: CV: cardiovascular; RASIs: renin-angiotensin system inhibitors

* Mediators-controlled hazard ratio (controlled direct effect): including systemic corticosteroids (excepting stratification by corticosteroids), anticoagulants and immunomodulators

Figure S7: Forest plots showing mortality rates and adjusted hazard ratios of in-hospital death associated with ACEIs discontinuation vs continuation and ARBs discontinuation vs continuation, by different subgroups (the category of reference is ARBs or ACEIs continued).

	ACEIs discontinued Deaths (%)	ACEIs continued Deaths (%)	MC−HR (95%CI)*	Test of interaction p−value
**RISK FACTORS:**				
**GENDER**				
Females	19 (32.2)	16 (29.1)	0.85 (0.43−1.68)	0.56
Males	27 (24.3)	29 (35.8)	0.62 (0.28−1.38)	
**AGE (years)**				
< 75	11 (12.1)	12 (18.2)	0.96 (0.35−2.62)	0.65
75+	35 (44.3)	33 (47.1)	0.73 (0.40−1.34)	
**OBESITY**				
No	38 (27.1)	33 (33.3)	0.70 (0.38−1.26)	0.86
Yes	8 (26.7)	12 (32.4)	0.62 (0.18−2.19)	
**DIABETES**				
No	34 (29.8)	31 (33.0)	0.73 (0.40−1.35)	0.62
Yes	12 (21.4)	14 (33.3)	0.53 (0.18−1.60)	
**HEART FAILURE**				
No	40 (25.5)	33 (28.5)	0.81 (0.45−1.44)	0.15
Yes	6 (46.2)	12 (60.0)	0.24 (0.05−1.09)	
**BACKGROUND CV RISK**				
CV risks factors	26 (23.9)	23 (29.1)	0.76 (0.37−1.52)	0.78
CV diseases	20 (32.8)	22 (38.6)	0.65 (0.28−1.51)	
**SEVERITY SCORE**				
0 − 3	25 (22.9)	25 (27.5)	0.62 (0.31−1.25)	0.59
4 − 7	21 (34.4)	20 (44.4)	0.77 (0.36−1.66)	
**IN−HOSPITAL USE OF:**				
**CORTICOSTEROIDS**				
No	27 (30.7)	22 (24.2)	0.88 (0.43−1.82)	0.16
Yes	19 (23.2)	23 (51.1)	0.40 (0.17−0.90)	
**BETA BLOCKERS**				
No	38 (26.4)	35 (31.0)	0.74 (0.42−1.32)	0.58
Yes	8 (30.8)	10 (43.5)	0.50 (0.14−1.79)	

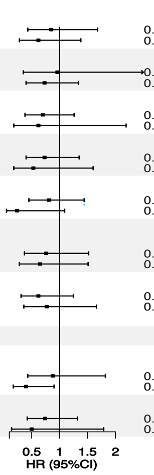

	ARBs discontinued Deaths (%)	ARBs continued Deaths (%)	MC−HR (95%CI)*	Test of interaction p−value
**RISK FACTORS:**				
**GENDER**				
Females	17 (25.4)	8 (14.0)	2.68 (0.82−8.78)	0.42
Males	31 (30.7)	18 (26.5)	1.51 (0.74−3.09)	
**AGE (years)**				
< 75	10 (12.7)	5 (7.9)	1.43 (0.38−5.30)	0.84
75+	38 (42.7)	21 (33.9)	1.67 (0.84−3.31)	
**OBESITY**				
No	36 (25.5)	19 (22.9)	1.30 (0.66−2.55)	0.08
Yes	12 (44.4)	7 (16.7)	5.40 (1.25−23.3)	
**DIABETES**				
No	21 (20.8)	13 (18.8)	1.35 (0.51−3.57)	0.74
Yes	27 (40.3)	13 (23.2)	1.67 (0.78−3.61)	
**HEART FAILURE**				
No	40 (26.3)	19 (17.8)	1.73 (0.88−3.42)	0.57
Yes	8 (50.0)	7 (38.9)	1.02 (0.19−5.50)	
**BACKGROUND CV RISK**				
CV risks factors	23 (20.0)	12 (15.6)	1.53 (0.65−3.61)	0.99
CV diseases	25 (47.2)	14 (29.2)	1.54 (0.64−3.71)	
**SEVERITY SCORE**				
0 − 3	25 (23.8)	15 (17.2)	1.75 (0.79−3.89)	0.84
4 − 7	23 (36.5)	11 (29.0)	1.54 (0.59−4.01)	
**IN−HOSPITAL USE OF:**				
**CORTICOSTEROIDS**				
No	16 (19.3)	8 (12.5)	1.54 (0.48−4.99)	0.99
Yes	32 (37.7)	18 (29.5)	1.53 (0.77−3.04)	
**BETA BLOCKERS**				
No	34 (25.2)	17 (19.3)	1.41 (0.69−2.89)	0.81
Yes	14 (42.4)	9 (24.3)	1.18 (0.34−4.10)	

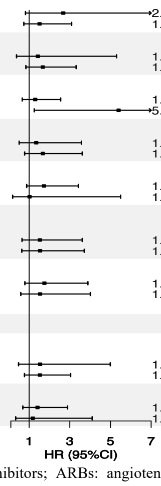

Abbreviations: ACEIs: angiotensin-converting enzyme inhibitors; ARBs: angiotensin receptor blockers; MC-HR: mediators-controlled hazard ratio; CV: cardiovascular

* Mediators-controlled hazard ratio (controlled direct effect): including systemic corticosteroids (except when stratification by corticosteroids), anticoagulants and immunomodulators

Figure S8: Density plot of estimated propensity scores according to discontinuation or continuation of RASIs. The probability of being discontinued with RASIs varied widely between hospital and date of admission. When those were included as covariates, the PS model captured such variability making both continuers and discontinuers very separable (panel a). If hospital and date of admission were not included in the model, the PS densities greatly overlap due to the fact that overall probability of being discontinued with RASIs was close to 0.5 and that made both discontinuers and continuers very comparable in their baseline characteristics (panel b).

a)

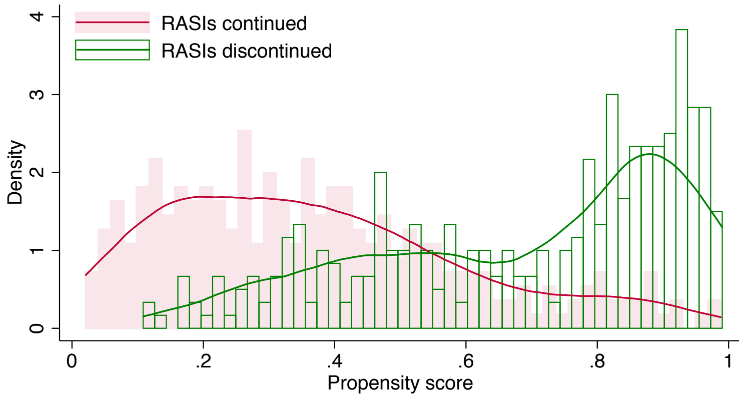

b)

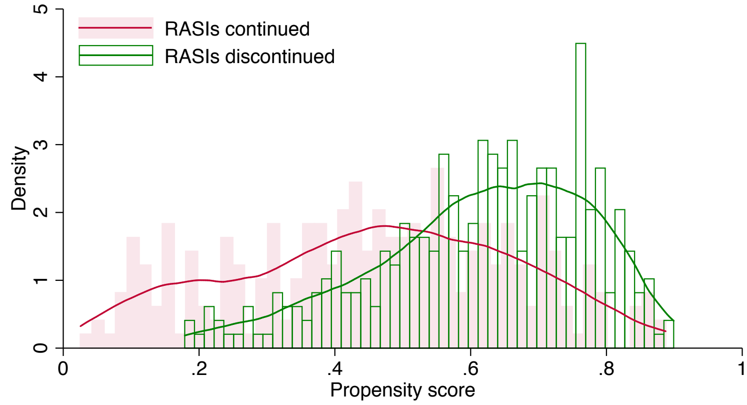

Table S1. Characteristics of patients who discontinued vs those who continued with ACEIs. All were users of ACEIs prior to admission. Baseline comorbidities, outpatient medications and intrahospital treatment

	ACEIs discontinued n=170 (%)	ACEIs continued n=136 (%)	Standardized difference	p-value
	**Baseline characteristics**			
**Sex,** males	111 (65.3)	81 (59.6)	+0.12	0.30
**Age,** years, median (IQR)	73 (63-82)	75 (68-82)	-0.17	0.26
**Hypertension**	166 (97.6)	133 (97.8)	0.01	0.93
**Obesity**	30 (17.6)	37 (27.2)	-0.23	**0.05**
**Smoking:**				
Non-smoker	61 (35.9)	54 (39.7)	-	0.51
Current smoker	9 (5.3)	7 (5.1)		
Past smoker	51 (30.0)	46 (33.8)		
Not recorded	49 (28.8)	29 (21.3)		
**Diabetes**	56 (32.9)	42 (30.9)	+0.04	0.70
**Dyslipidemia**	112 (65.9)	96 (70.6)	-0.10	0.38
**CV risk:**				
CV risk factors	109 (64.1)	79 (58.1)	+0.12	0.28
History of CV disease	61 (35.9)	57 (41.9)		
**COPD**	18 (10.6)	19 (14.0)	-0.10	0.37
**Asthma**	13 (7.6)	7 (5.1)	+0.10	0.38
**Cancer:**				
Antecedents	19 (11.2)	15 (11.0)	+0.005	0.97
Current	22 (12.9)	14 (10.3)	+0.08	0.48
**Chronic renal failure**	18 (10.6)	13 (9.6)	+0.03	0.77
**Outpatient medication**:				
AMRs	5 (2.9)	6 (4.4)	-0.08	0.49
CCBs	50 (29.4)	26 (19.1)	+0.24	**0.04**
Diuretics	74 (43.5)	60 (44.1)	+0.01	0.92
Beta-blockers	39 (22.9)	28 (20.6)	+0.06	0.62
Alpha-blockers	6 (3.5)	7 (5.1)	-0.08	0.49
Oral anticoagulants	24 (14.1)	29 (21.3)	-0.19	0.10
Antiplatelet agents	47 (27.6)	43 (31.6)	-0.09	0.45
NSAIDs	16 (9.4)	8 (5.9)	+0.13	0.25
Corticosteroids	15 (8.8)	3 (2.2)	+0.29	**0.01**
Paracetamol	87 (51.2)	81 (59.6)	-0.17	0.14
Metamizole	37 (21.8)	38 (27.9)	-0.14	0.21
Statins	91 (53.5)	81 (59.6)	-0.12	0.29
Ezetimibe	8 (4.7)	3 (2.2)	+0.14	0.24
Glucose lowering drugs	47 (27.6)	35 (25.7)	+0.04	0.71
Insulin	12 (7.1)	13 (9.6)	-0.09	0.43
**Pneumonia**	159 (93.5)	117 (86.0)	+0.25	**0.03**

**Severity score**				
0-1	20 (11.8)	20 (14.7)		
**2**	34 (20.0)	30 (22.1)		
**3**	55 (32.4)	41 (30.1)	-	0.88
**4**	44 (25.9)	36 (26.5)		
**5**	13 (7.6)	7 (5.1)		
**6-7**	4 (2.4)	2 (1.5)		
**Mean (SD)**	3.0 (1.2)	2.9 (1.2)	+0.12	0.30
**In-hospital stay and treatments received in hospital**				
**Stay, days, median (IQR)**	11 (7-17)	10 (6-15.5)	+0.05	0.23
**ICU admission**	9 (5.3)	7 (5.1)	0.01	0.95
**Anticoagulants**:				
Oral	12 (7.1)	20 (14.7)	-0.25	**0.03**
Parenteral	136 (80.0)	79 (58.1)	+0.49	**<0.001**
**Antiplatelet drugs**	43 (25.3)	43 (31.6)	-0.14	0.22
**Statins**	27 (15.9)	59 (43.4)	-0.63	**<0.001**
**Glucose lowering drugs**:				
Oral	7 (4.1)	19 (14.0)	-0.35	**0.002**
Insulin	56 (32.9)	38 (27.9)	+0.11	0.35
**Hydroxychloroquine**	151 (88.8)	115 (84.6)	+0.13	0.27
**Lopinavir+Ritonavir/ Darunavir+Cobicistat**	142 (83.5)	109 (80.1)	+0.09	0.44
**Azithromycin**	78 (45.9)	52 (38.2)	+0.15	0.18
**Other macrolides**	3 (1.8)	7 (5.1)	-0.19	0.10
**Other antivirals***	3 (1.8)	5 (3.7)	-0.12	0.30
**Other antibacterial agents**	113 (66.5)	92 (67.6)	-0.02	0.83
**Immunomodulating agents**:				
Tocilizumab	22 (12.9)	30 (22.1)	-0.24	**0.04**
Others**	53 (31.2)	59 (43.4)	-0.25	**0.03**
**Corticosteroids**	82 (48.2)	45 (33.1)	+0.31	**0.01**
**Antihypertensive drugs**:				
CCBs	66 (38.8)	29 (21.3)	+0.39	**0.001**
Beta-blockers	26 (15.3)	23 (16.9)	-0.04	0.70
Low-ceiling diuretics	6 (3.5)	14 (10.3)	-0.27	**0.02**
High-ceiling diuretics	26 (15.3)	22 (16.2)	+0.02	0.83
Alpha-blockers	2 (1.2)	3 (2.2)	-0.08	0.48

Abbreviations: ACEIs: angiotensin-converting enzyme inhibitors; IQR: interquartile range; CV: cardiovascular; COPD: chronic obstructive pulmonary disease; AMRs: antagonists of mineralocorticoid receptor; CCBs: calcium channel blockers NSAIDs: nonsteroidal anti-inflammatory drugs; SD: standard deviation; ICU: intensive care unit

* Other antivirals: remdesivir, aciclovir, bictegravir-emtricitabine-tenofovir, tenofovir, emtricitabine-tenofovir, lamivudine-abacabir-dolutegravir, valaciclovir and valganciclovir.

** Other immunomodulators: Jak inhibitors, interferon beta-1b, ciclosporin, anakinra, ceftriaxone, leflunomide, methotrexate and mycophenolic acid.

Table S2. Characteristics of patients who discontinued vs those who continued with ARBs. All were users of ARBs prior to admission. Baseline comorbidities, outpatient medications and intrahospital treatment

	ARBs discontinued n=168 (%)	ARBs continued n=125 (%)	Standardized difference	p-value
	**Baseline characteristics**			
**Sex,** males	101 (60.1)	68 (54.4)	+0.12	0.33
**Age,** years, median (IQR)	75 (67-82)	74 (69-82)	+0.12	0.74
**Hypertension**	164 (97.6)	120 (96.0)	+0.09	0.43
**Obesity**	27 (16.1)	42 (33.6)	-0.41	**<0.001**
**Smoking:**				
Non-smoker	66 (39.3)	52 (41.6)		
Current smoker	5 (3.0)	6 (4.8)		
Past smoker	51 (30.4)	30 (24.0)	-	0.59
Not recorded	46 (27.4)	37 (29.6)		
**Diabetes**	67 (39.9)	56 (44.8)	-0.10	0.40
**Dyslipidemia**	106 (63.1)	80 (64.0)	-0.02	0.87
**CV risk:**				
CV risk factors	115 (68.5)	77 (61.6)	+0.14	0.22
**History of CV disease**	53 (31.5)	48 (38.4)		
**COPD**	24 (14.3)	13 (10.4)	+0.12	0.32
**Asthma**	14 (8.3)	11 (8.8)	+0.02	0.89
**Cancer:**				
Antecedents	13 (7.7)	13 (10.4)	-0.09	0.43
Current	13 (7.7)	12 (9.6)	+0.07	0.57
**Chronic Renal Failure**	20 (11.9)	18 (14.4)	-0.07	0.53
**Baseline co-medication**:				
AMRs	6 (3.6)	6 (4.8)	-0.06	0.60
CCBs	64 (38.1)	48 (38.4)	-0.01	0.96
Diuretics	98 (58.3)	80 (64.0)	-0.12	0.33
Beta-blockers	41 (24.4)	37 (29.6)	-0.12	0.32
Alpha-blockers	14 (8.3)	12 (9.6)	-0.04	0.71
Oral anticoagulants	31 (18.5)	22 (17.6)	+0.02	0.85
Antiplatelet agents	45 (26.8)	33 (26.4)	+0.01	0.94
NSAIDs	13 (7.7)	9 (7.2)	+0.02	0.86
Corticosteroids	10 (6.0)	7 (5.6)	+0.02	0.90
Paracetamol	89 (53.0)	80 (64.0)	-0.22	0.06
Metamizole	57 (33.9)	33 (26.4)	+0.16	0.17
Statins	81 (48.2)	69 (55.2)	-0.14	0.24
Ezetimibe	6 (3.6)	9 (7.2)	+0.16	0.16
Glucose lowering drugs	53 (31.5)	50 (40.0)	-0.18	0.13
Insulin	21 (11.9)	20 (16.0)	-0.12	0.31
**Pneumonia**	158 (94.1)	115 (92.0)	+0.09	0.49

**Severity score**				
0-1	19 (11.3)	15 (12.0)		
2	32 (19.0)	42 (33.6)	-	0.05
3	54 (32.1)	30 (24.0)		
4	34 (20.2)	26 (20.8)		
5	26 (15.5)	10 (8.0)		
6-7	3 (1.8)	2 (1.6)		
Mean (SD)	3.2 (1.3)	2.8 (1.2)	+0.25	**0.03**
**In-hospital stay and treatments received in hospital**				
**Stay,** days, median (IQR)	11 (8-17)	12 (7-16)	-0.04	0.79
**ICU admission**	10 (6.0)	9 (7.2)	+0.05	0.67
**Anticoagulants**:				
Oral	14 (8.3)	16 (12.8)	-0.15	0.21
Parenteral	135 (80.4)	69 (55.2)	+0.56	**<0.001**
**Antiplatelet drugs**	36 (21.4)	37 (29.6)	-0.19	0.11
**Statins**	19 (11.3)	50 (40.0)	-0.69	**<0.001**
**Glucose lowering drugs**:				
Oral	7 (4.2)	22 (17.6)	-0.44	**<0.001**
Insulin	69 (41.1)	40 (32.0)	+0.19	0.11
**Hydroxychloroquine**	153 (91.1)	108 (86.4)	+0.15	0.20
**Lopinavir+Ritonavir/ Darunavir+Cobicistat**	143 (85.1)	104 (83.2)	+0.05	0.66
**Azithromycin**	50 (29.8)	52 (41.6)	-0.25	**0.04**
**Other macrolides**	7 (4.2)	10 (8.0)	-0.16	0.17
**Other antivirals***	5 (3.0)	1 (0.8)	+0.16	0.19
**Other antibacterial agents**	98 (58.3)	85 (68.0)	-0.20	0.09
**Immunomodulating agents**:				
Tocilizumab	21 (12.5)	23 (18.4)	-0.16	0.16
Others**	45 (26.8)	54 (43.2)	-0.35	**0.003**
**Corticosteroids**	85 (50.6)	61 (48.8)	+0.04	0.76
**Antihypertensive drugs**:				
CCBs	64 (38.1)	36 (28.8)	+0.20	0.10
Beta-blockers	33 (19.6)	37 (29.6)	-0.23	0.05
Low-ceiling diuretics	11 (6.5)	33 (26.4)	-0.55	**<0.001**
High-ceiling diuretics	25 (14.9)	14 (11.2)	+0.11	0.36
Alpha-blockers	9 (5.4)	6 (4.8)	+0.03	0.83

Abbreviations: ACEIs: angiotensin-converting enzyme inhibitors; IQR: interquartile range; CV: cardiovascular; COPD: chronic obstructive pulmonary disease; AMRs: antagonists of mineralocorticoid receptor; CCBs: calcium channel blockers NSAIDs: nonsteroidal anti-inflammatory drugs; SD: standard deviation; ICU: intensive care unit

* Other antivirals: remdesivir, aciclovir, bictegravir-emtricitabine-tenofovir, tenofovir, emtricitabine-tenofovir, lamivudine-abacabir-dolutegravir, valaciclovir and valganciclovir.

** Other immunomodulators: Jak inhibitors, interferon beta-1b, ciclosporin, anakinra, ceftriaxone, leflunomide, methotrexate and mycophenolic acid.

Table S3. Characteristics of patients who continued with ACEIs as compared to those who continued with ARBs. All patients were prior users of ACEIs or ARBs. Baseline comorbidities, outpatient medication and intrahospital treatment.

	ACEIs continued n=136 (%)	ARBs continued n=125 (%)	Standardized difference	p-value
	**Baseline characteristics**			
**Sex, m**ales	81 (59.6)	68 (54.4)	+0.10	0.40
**Age,** years, median (IQR)	75 (68-82)	74 (69-82)	+0.09	0.89
**Hypertension**	133 (97.8)	120 (96.0)	+0.10	0.40
**Obesity**	37 (27.2)	42 (33.6)	-0.14	0.26
**Smoking:**				
Non-smoker	54 (39.7)	52 (41.6)		
Current smoker	7 (5.1)	6 (4.8)	-	0.26
Past smoker	46 (33.8)	30 (24.0)		
Not recorded	29 (21.3)	37 (29.6)		
**Diabetes**	42 (30.9)	56 (44.8)	-0.29	**0.02**
**Dyslipidemia**	96 (70.6)	80 (64.0)	+0.14	0.26
**CV risk:**				
CV risk factors	79 (58.1)	77 (61.6)	-0.07	0.56
History of CV disease	57 (41.9)	48 (38.4)		
**COPD**	19 (14.0)	13 (10.4)	+0.11	0.38
**Asthma**	7 (5.1)	11 (8.8)	-0.14	0.24
**Cancer:**				
Antecedents	15 (11.0)	13 (10.4)	+0.02	0.87
Current	14 (10.3)	12 (9.6)	+0.02	0.85
**Chronic Renal Failure**	13 (9.6)	18 (14.4)	-0.15	0.23
**Medication before admission**:				
AMRs	6 (4.4)	6 (4.8)	-0.02	0.88
CCBs	26 (19.1)	48 (38.4)	-0.43	**<0.001**
Diuretics	60 (44.1)	80 (64.0)	-0.41	**0.001**
Beta-blockers	28 (20.6)	37 (29.6)	-0.21	0.09
Alpha-blockers	7 (5.1)	12 (9.6)	-0.17	0.17
Oral anticoagulants	29 (21.3)	22 (17.6)	+0.09	0.45
Antiplatelet agents	43 (31.6)	33 (26.4)	+0.11	0.35
NSAIDs	8 (5.8)	9 (7.1)	-0.05	0.65
Corticosteroids	3 (2.2)	7 (5.6)	-0.18	0.15
Paracetamol	81 (59.6)	80 (64.0)	-0.09	0.46
Metamizole	38 (27.9)	33 (26.4)	+0.03	0.78
Statins	81 (59.6)	69 (55.2)	+0.09	0.48
Ezetimibe	3 (2.2)	9 (7.2)	-0.24	0.05
Glucose lowering drugs	35 (25.7)	50 (40.0)	-0.31	**0.01**
Insulin	13 (9.6)	20 (16.0)	-0.19	0.12
**Pneumonia**	117 (86.0)	115 (92.0)	-0.19	0.13

**Severity score**	20 (14.7)	15 (12.0)		
0-1	30 (22.1)	42 (33.6)		
2	41 (30.1)	30 (24.0)		
3	36 (26.5)	26 (20.8)	-	0.30
4	7 (5.1)	10 (8.0)		
5	2 (1.5)	2 (1.6)		
6-7	2.9 (1.2)	2.8 (1.2)	0.05	0.66
Mean (SD)				
**In-hospital stay and treatments received in hospital**				
**Stay,** days, median (IQR**)**	10 (6-15.5)	12 (7-16)	-0.14	0.17
**ICU admission**	7 (5.1)	9 (7.2)	-0.09	0.49
**Anticoagulants**:				
Oral	20 (14.7)	16 (12.8)	+0.06	0.66
Parenteral	79 (58.1)	69 (55.2)	+0.06	0.64
**Antiplatelet drugs**	43 (31.6)	37 (29.6)	+0.04	0.72
**Statins**	59 (43.4)	50 (40.0)	+0.07	0.58
**Glucose lowering drugs:**				
Oral	19 (14.0)	22 (17.6)	-0.10	0.42
Insulin	38 (27.9)	40 (32.0)	+0.09	0.47
**Hydroxychloroquine**	115 (84.6)	108 (86.4)	-0.05	0.67
**Lopinavir+Ritonavir/ Darunavir+Cobicistat**	109 (80.1)	104 (83.2)	-0.08	0.52
**Azithromycin**	52 (38.2)	52 (41.6)	-0.07	0.58
**Other macrolides**	7 (5.1)	10 (8.0)	-0.11	0.35
**Other antivirals***	5 (3.7)	1 (0.8)	+0.19	0.12
**Other antibacterial agents**	92 (67.6)	85 (68.0)	0.01	0.95
**Immunomodulating agents**:				
Tocilizumab	30 (22.1)	23 (18.4)	+0.09	0.46
Others**	59 (43.4)	54 (43.2)	+0.004	0.98
**Corticosteroids**	45 (33.1)	61 (48.8)	-0.32	**0.01**
**Antihypertensive drugs**:				
CCBs	29 (21.3)	36 (28.8)	-0.17	0.16
Beta-blockers	23 (16.9)	37 (29.6)	-0.30	**0.02**
Low-ceiling diuretics	14 (10.3)	33 (26.4)	-0.42	**<0.001**
High-ceiling diuretics	22 (16.2)	14 (11.2)	+0.14	0.24
Alpha-blockers	3 (2.2)	6 (4.8)	-0.14	0.25

Abbreviations: ACEIs: angiotensin-converting enzyme inhibitors; ARBs: angiotensin receptor blockers; IQR: interquartile range; CV: cardiovascular; COPD: chronic obstructive pulmonary disease; AMRs: antagonists of mineralocorticoid receptor; CCBs: calcium channel blockers NSAIDs: nonsteroidal anti-inflammatory drugs; SD: standard deviation; ICU: intensive care unit

* Other antivirals: remdesivir, aciclovir, bictegravir-emtricitabine-tenofovir, tenofovir, emtricitabine-tenofovir, lamivudine-abacabir-dolutegravir, valaciclovir and valganciclovir.

** Other immunomodulators: Jak inhibitors, interferon beta-1b, ciclosporin, anakinra, ceftriaxone, leflunomide, methotrexate and mycophenolic acid.

Table S4: Sensitivity analyses (the category of reference is RASIs, ARBs or ACEIs continued)

1. Reclassification of uncertain to RASIs discontinued if they had a sole prescription on day 1 and to RASIs continued if they had a sole prescription on days 2 or 3.

Outcome	RASIs discontinued N=362		RASIs continued N=316		Crude HR (95%CI)	PS-adj HR* (95%CI)	MC-HR** (95%CI)
	Patients with event	Event rate (%)	Patients with event	Event rate (%)			
Death	100	27.6	84	26.6	1.02 (0.77-1.37)	1.15 (0.82-1.60)	1.16 (0.83-1.62)
Death + ICU	115	31.8	95	30.1	1.03 (0.79-1.35)	1.17 (0.86-1.61)	1.22 (0.89-1.67)

2. Reclassification of all uncertain to the cohort of patients who discontinued

Outcome	RASIs discontinued N=393		RASIs continued N=285		Crude HR (95%CI)	PS-adj HR* (95%CI)	MC-HR** (95%CI)
	Patients with event	Event rate (%)	Patients with event	Event rate (%)			
Death	105	26.7	79	27.7	0.90 (0.68-1.21)	0.98 (0.69-1.39)	0.99 (0.70-1.41)
Death + ICU	121	30.8	89	31.2	0.93 (0.70-1.22)	1.02 (0.74-1.42)	1.06 (0.76-1.48)

3. Using a 2-days window (uncertain excluded)

Outcome	RASIs discontinued N=358		RASIs continued N=227		Crude HR (95%CI)	PS-adj HR* (95%CI)	MC-HR** (95%CI)
	Patients with event	Event rate (%)	Patients with event	Event rate (%)			
Death	98	27.4	65	28.6	0.90 (0.66-1.23)	0.87 (0.59-1.30)	0.90 (0.60-1.35)
Death + ICU	112	31.3	73	32.2	0.91 (0.68-1.22)	0.93 (0.63-1.35)	0.96 (0.66-1.40)

Outcome	ARBs discontinued N=177		ARBs continued N=100		Crude HR (95%CI)	PS-adj HR* (95%CI)	MC-HR** (95%CI)
	Patients with event	Event rate (%)	Patients with event	Event rate (%)			
Death	50	28.3	21	21.0	1.33 (0.80-2.22)	1.85 (0.95-3.61)	1.84 (0.94-3.61)
Death + ICU	57	32.2	26	26.0	1.21 (0.76-1.93)	1.32 (0.70-2.50)	1.41 (0.73-2.70)

Outcome	ACEIs discontinued N=179		ACEIs continued N=115		Crude HR (95%CI)	PS-adj HR* (95%CI)	MC-HR** (95%CI)
	Patients with event	Event rate (%)	Patients with event	Event rate (%)			
Death	48	26.8	38	33.0	0.72 (0.47-1.11)	0.67 (0.38-1.17)	0.69 (0.38-1.22)
Death + ICU	55	30.7	41	35.7	0.77 (0.51-1.15)	0.78 (0.46-1.33)	0.81 (0.48-1.37)

4. Using a 2-days window (uncertain reclassified to RASIs discontinued if they had a sole prescription on day 1 and to RASIs continued if had a sole prescription on day 2)

**Outcome**	**RASIs discontinued N=380**		**RASIs continued N=298**		**Crude HR (95%CI)**	**PS-adj HR* (95%CI)**	**MC-HR** (95%CI)**
	**Patients with event**	**Event rate (%)**	**Patients with event**	**Event rate (%)**			
**Death**	104	27.4	80	26.9	0.97 (0.73-1.30)	1.09 (0.77-1.54)	1.10 (0.78-1.56)
**Death + ICU**	120	31.6	90	30.2	1.01 (0.77-1.32)	1.15 (0.83-1.59)	1.17 (0.85-1.62)

**Outcome**	**ARBs discontinued N=188**		**ARBs continued N=131**		**Crude HR (95%CI)**	**PS-adj HR* (95%CI)**	**MC-HR** (95%CI)**
	**Patients with event**	**Event rate (%)**	**Patients with event**	**Event rate (%)**			
**Death**	53	28.2	27	20.6	1.32 (0.83-2.10)	1.74 (0.97-3.12)	1.88 (1.06-3.34)
**Death + ICU**	60	31.9	33	25.2	1.21 (0.79-1.85)	1.39 (0.81-2.41)	1.51 (0.87-2.61)

**Outcome**	**ACEIs discontinued N=190**		**ACEIs continued N=144**		**Crude HR (95%CI)**	**PS-adj HR* (95%CI)**	**MC-HR** (95%CI)**
	**Patients with event**	**Event rate (%)**	**Patients with event**	**Event rate (%)**			
**Death**	51	26.8	45	31.3	0.81 (0.55-1.22)	0.77 (0.48-1.23)	0.78 (0.48-1.26)
**Death + ICU**	60	31.6	48	33.3	0.92 (0.63-1.35)	0.98 (0.62-1.54)	0.98 (0.63-1.54)

Abbreviations: RASIs: renin-angiotensin system inhibitors; HR: hazard ratio; ICU: intensive care unit
*Propensity-scores-adjusted hazard ratio (adjusted total effect)
** Mediators-controlled hazard ratio (controlled direct effect): a) systemic corticosteroids, anticoagulants and immunomodulators when the outcome was death; and b) immunomodulators and anticoagulants when the outcome was death plus ICU admission

Table S5: Published studies reporting mortality rates associated with inpatient use of renin-angiotensin system inhibitors (RASI)

First Author	Country	Definition of inpatient RASI use (or cont)	Primary outcome	Number of inpatients	Disc rate	Main results Adjusted HR/OR (95%CI)
Cannata [9]	Italy	At the time of hospital admission	Mortality	56 cont RASIs<br>117 disc RASIs<br>224 no RASIs	67.7%	RASI cont vs disc:<br>0.17 (0.03-1.00)<br>RASI cont vs no RASI + disc: **0.14 (0.03-0.66)**
Chaudhri [12]	US	Any time during hospitalization	In-hospital mortality	49 cont RASIs<br>31 disc RASIs	38.8%	RASI cont vs disc:<br>0.31 (0.08-1.26)
Chen [32]	China	Any time during hospitalization	In-hospital mortality	93 RASIs<br>2530 no RASIs<br>53 RASIs<br>315 non-RASIs	Not reported	RASI vs. no RASI:<br>0.40 (0.15-1.03)<br>RASI vs. non-RASI:<br>**0.18 (0.04-0.86)**
Di Castelnuovo (CORIST) [28]	Italy	Any time during hospitalization	In-hospital mortality	549 ACEIs<br>542 ARBs<br>2807 no RASIs	12.4%	ACEIs vs no RASI:<br>0.96 (0.77-1.20);<br>ARBs vs. no RASI:<br>0.89 (0.71-1.12)
Lahens [17]	France	Previous users who continued during the first 7 days of hospital admission	30-day mortality	84 RASIs<br>263 no RASIs	33.0%	RASI vs non-RASI:<br>**0.25 (0.09-0.65)**
Lam [10]	US	Any time during hospitalization	In-hospital mortality	164 cont RASIs<br>171 disc RASIs	51.0%	RASI cont vs disc:<br>**0.215 (0.101-0.455)**
Li [27]	China	Use at time of admission that continued through hospitalization	In-hospital mortality	115 RASIs<br>247 non-RASIs	Not reported	HR/OR not reported<br>RASI: 18.3%<br>No-RASI: 22.7%
Meng [34]	China	Any time during hospitalization	In-hospital mortality	17 RASIs<br>25 non-RASIs	Not reported	HR/OR not reported<br>RASI: 0.0%<br>No-RASI: 4.0%
Rodilla [30]	Spain	Any time during hospitalization	In-hospital mortality	ACEIs: 490<br>ARBs: 1046	ACEIs: 55.1%<br>ARBs: 53.6%	In-hospital ACEIs vs non-use: **0.6 (0.45-0.66)**<br>In-hospital ARBs vs non-use: **0.5 (0.45-0.65)**
Soleimani [31]	Iran	During 7 days after admission	In-hospital mortality	ARBs:<br>79 cont<br>43 disc	35.2%	HR/OR not reported<br>ARB cont: 12.7%<br>ARB disc: 53.5%
Wang [29]	China	Any time during hospitalization	Mortality	81 RASIs<br>129 non-RASIs	Not reported	HR/OR not reported<br>RASI: 8.64%<br>Non-RASI: 3.88%
Zhang [11]	China	Any time during hospitalization	28-day all-cause mortality	188 RASIs<br>940 no RASIs	Not reported	RASI vs non-RASI:<br>**0.42 (0.19-0.92)**
Zhou [33]	China	Any time during hospitalization	28-day all-cause mortality	989 RASIs<br>2583 no RASIs	Not reported	RASI vs non-RASI:<br>**0.39 (0.26-0.58)**

Abbreviations: ACEIs: angiotensin converting enzyme inhibitors; ARBs: angiotensin receptor blocker; Cont: continuation; Disc: discontinuation; Disc rate: discontinuation rate; HR: hazard ratio; ICU: intensive care unit; OR: odds ratio; RASIs: renin-angiotensin system inhibitors
Note: In this table, “non-RASIs” means that patients received other antihypertensive drugs different from RASIs, while “no RASIs” means that patients were not exposed to RASIs (but not necessarily exposed to other antihypertensive drugs). Statistically significant HR/OR values are in bold.
