# Supplementary material for: Impact of in-hospital discontinuation with angiotensin receptor blockers or converting enzyme inhibitors on mortality of COVID-19 patients: a retrospective cohort study
Source: BMC Med. 2021 May 12;19:118. doi: 10.1186/s12916-021-01992-9 (PMC8114973; doi:10.1186/s12916-021-01992-9)
Supplement: Supplementary file 2 — Additional file 2. Graphical abstract. [file 12916_2021_1992_MOESM2_ESM.pdf]

IMPACT OF IN-HOSPITAL DISCONTINUATION OF ANGIOTENSIN RECEPTOR BLOCKERS OR ANGIOTENSIN CONVERTING ENZYME INHIBITORS ON MORTALITY OF COVID-19 PATIENTS:
A RETROSPECTIVE COHORT STUDY

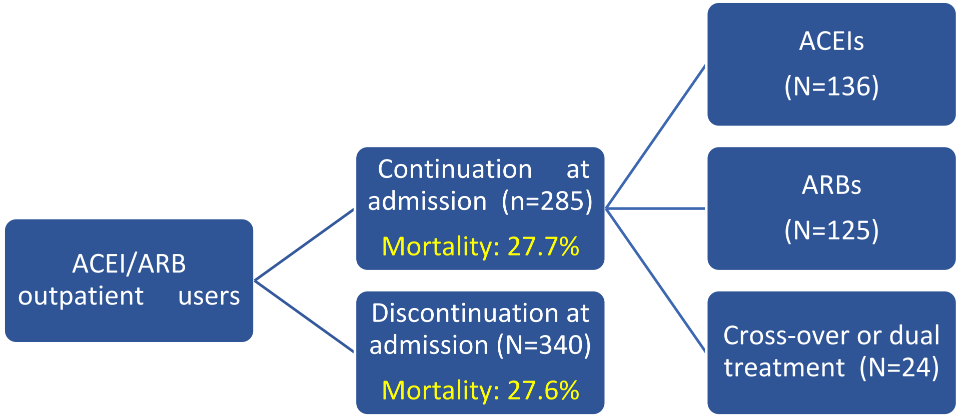

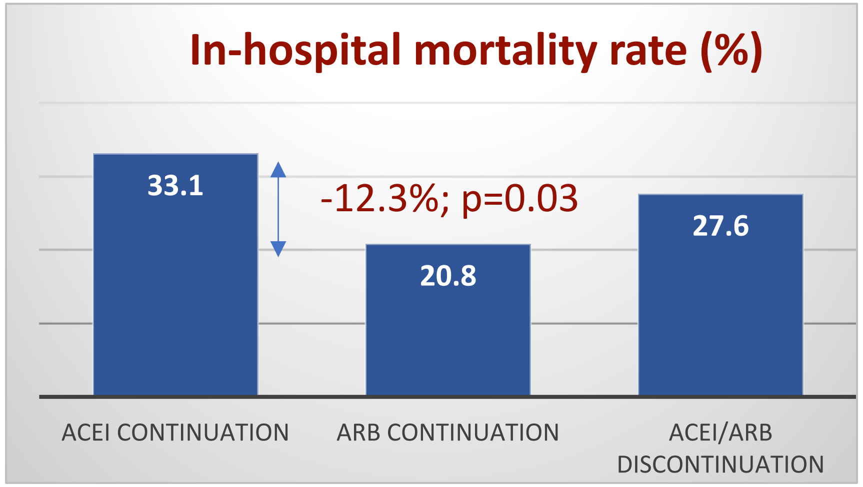

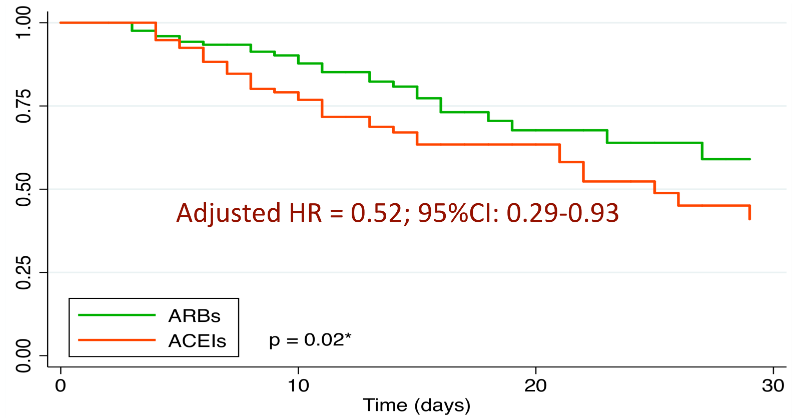

Number at risk:				
ACEIs	136	70	26	9
ARBs	125	75	21	9

* Logrank test

	ARBs continued Deaths (%)	ACEIs continued Deaths (%)	MC–HR (95%CI)*	Test of interaction p–value
**RISK FACTORS:**				
**GENDER**				
Females	8 (14.0)	16 (29.1)	0.58 (0.27–1.26)	0.40
Males	18 (26.5)	29 (35.8)	0.34 (0.13–0.93)	
**AGE (years)**				
< 75	5 (7.9)	12 (18.2)	0.42 (0.11–1.66)	0.91
75+	21 (33.9)	33 (47.1)	0.46 (0.25–0.85)	
**OBESITY**				
No	19 (22.9)	33 (33.3)	0.56 (0.28–1.10)	0.26
Yes	7 (16.7)	12 (32.4)	0.22 (0.05–0.94)	
**DIABETES**				
No	13 (18.8)	31 (33.0)	0.56 (0.26–1.20)	0.49
Yes	13 (23.2)	14 (33.3)	0.36 (0.13–0.97)	
**HEART FAILURE**				
No	19 (17.8)	33 (28.5)	0.63 (0.31–1.26)	0.04
Yes	7 (38.9)	12 (60.0)	0.12 (0.03–0.48)	
**BACKGROUND CV RISK**				
CV risks factors	12 (15.6)	23 (29.1)	0.59 (0.25–1.39)	0.61
CV diseases	14 (29.2)	22 (38.6)	0.43 (0.18–1.03)	
**SEVERITY SCORE**				
0 – 3	15 (17.2)	25 (27.5)	0.59 (0.27–1.27)	0.73
4 – 7	11 (29.0)	20 (44.4)	0.48 (0.20–1.14)	

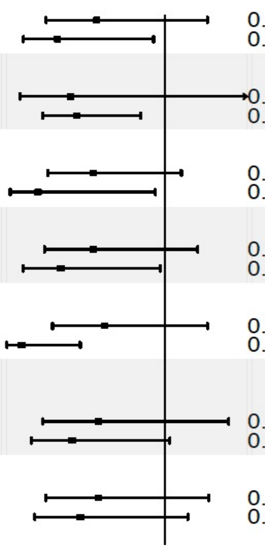

CONCLUSIONS: The discontinuation of ACEIs/ARBs at admission did not improve the in-hospital survival. On the contrary, the continuation of ARBs was associated with a trend to a reduced mortality as compared to their discontinuation and with a significantly lower mortality risk as compared to the continuation of ACEIs, especially in high-risk patients.
